# Patient-derived organoids in functional precision oncology: from experimental models to clinical decision-making

**DOI:** 10.3389/fendo.2026.1872361

**Published:** 2026-07-22

**Authors:** Amanda Caruso, A Delvecchio, R Memeo, M Lanzino, S Martinotti

**Affiliations:** 1 Department of Medicine and Surgery, LUM University "Giuseppe Degennaro", Casamassima, Bari, Italy; 2 Unit of Hepato-Biliary and Pancreatic Surgery, Ecclesiastical Entity Regional General Hospital “F. Miulli” General Hospital, Acquaviva delle Fonti, Bari, Italy; 3 Department of Pharmacy, Health and Nutritional Sciences, University of Calabria, Rende, Italy; 4 Clinical Pathology Unit, “F. Miulli” University Hospital, Acquaviva delle Fonti, Italy

**Keywords:** 3D cell culture, organoid-on-a-chip, patient-derived organoid (PDO), precision medicine & genomics, targeted therapy

## Abstract

Despite major advances in high-throughput genomics, proteomics, and multimodal imaging, a substantial gap persists between molecular tumor characterization and clinically actionable therapeutic decision-making, partly due to the limitations of conventional preclinical models in capturing tumor heterogeneity and predicting patient-specific drug response. Patient-derived organoids (PDO) have emerged as a promising platform to bridge this gap by enabling functional interrogation of individual tumors in a physiologically relevant three-dimensional context. PDO retain the genomic, transcriptomic, and histopathological features of their parental tumors while supporting long-term expansion, biobanking, and high-throughput pharmacological testing. In this review, we provide a clinically oriented overview of PDO technology as a key tool in functional precision oncology, summarizing current methodologies for tissue processing, organoid derivation, and quality control. We examine applications across multiple cancer types, including drug screening, radiotherapy response modeling, immuno-oncology co-culture systems, and CRISPR-based functional genomics, highlighting their role in directly measuring therapeutic vulnerability. We also integrate tumor-specific evidence across major malignancies, including colorectal, pancreatic, and breast cancers, where PDO-based pharmacotyping shows strong concordance with clinical outcomes and is increasingly incorporated into prospective trials. Finally, we discuss the integration of PDO with emerging technologies, including organoid-on-chip systems, artificial intelligence-driven analytics, and hospital-integrated workflows, as a critical innovation layer that is redefining their clinical applicability. These integrative approaches move PDO beyond static ex vivo models toward dynamic, and decision-support systems, with the potential to substantially enhance predictive accuracy and real-time therapeutic stratification. Collectively, these advances position PDOs as a promising component in next-generation precision oncology, supporting a transition from static genomics-based stratification toward dynamic, functionally guided therapeutic decision-making.

## 1 Introduction

Over the past decades, advances in high-throughput genomics have revealed the extensive molecular heterogeneity of cancer, highlighting the persistent translational gap between molecular insights and clinical application (1). A major contributor to this gap is the experimental models currently used. In fact, although the traditional two dimensional monolayers *in vitro* systems have been used for a long time in cancer research, with HeLa cells representing one of the earliest established models (2), they lack the spatial architecture, cellular diversity, and biochemical gradients that shape drug sensitivity *in vivo* (3–5). Subsequently, various three-dimensional (3D) cell culture approaches were developed. Early three dimensional spheroids offered incremental advances, recapitulating nutrient and oxygen diffusion gradients and supporting stem-like subclones, but often genetically and phenotypically different from the donor tumor (6). More sophisticated explant cultures and raft systems, such as spheroids, improve tissue fidelity but suffer from limited culture maintenance, lack of proliferation, and low establishment success rates, restricting their utility for systematic pharmacological interrogation (6–9). In this context, PDOs have emerged as a robust platform for cancer modeling. Building on foundational work by Clevers and colleagues, who demonstrated that single LGR5+ intestinal stem cells could generate crypt-villus structures *in vitro* (10), organoid technology exploits the self-organizing capacity of adult epithelial progenitors to recapitulate the cytoarchitecture and cellular heterogeneity of patient tumors. Unlike traditional cell lines or spheroids, PDO retain the molecular, histopathological, and functional features of their tumor of origin (1, 11). These models can be cultured long-term, cryopreserved, genetically manipulated, and used for high-throughput pharmacological testing (12–15). Moreover, the integration of PDO with gene editing platforms (e.g., CRISPR-Cas9), single-cell transcriptomics, and co-culture systems incorporating stromal or immune components offers an unprecedented resolution to interrogate tumor-intrinsic and microenvironmental dependencies (16). Parallel advances in artificial intelligence are accelerating each step, such as automate image based viability scoring, and fuse histology, genomics, and functional readouts into predictive models that can inform real time therapeutic decision making (17–23). Notably, recent updates to U.S. FDA regulatory frameworks have encouraged the adoption of human-relevant preclinical systems, including organoids, artificial intelligence–assisted models, and real-world data, with the broader aim of reducing reliance on animal testing (24). Thus, the concept of “PDO-enabled precision oncology” encapsulates a roadmap toward truly personalized therapy. In parallel with PDO development, organotypic tissue slice cultures (OTSCs) have emerged as complementary patient-derived models preserving the native tumor architecture, stromal compartment, and immune infiltrate (25). Unlike PDOs, OTSCs maintain the original tissue organization and allow rapid ex vivo drug testing (26). However, their limited viability and scalability restrict long-term expansion and high-throughput applications. Therefore, OTSCs and PDOs should be considered complementary platforms for precision oncology.This review charts the clinic-translational journey of PDO from bench to bedside. Particularly, we propose that PDO represent the central enabling technology for the transition from molecular to functional precision oncology.

We first outline state of the art methods for tissue procurement, organoid derivation, and biobanking; then survey tumor-agnostic applications—including high throughput chemotherapy and radiotherapy screens, gene editing studies, and complex TME platforms. We next synthesise tumor specific evidence across metastatic colorectal cancer, PDAC, breast cancer (BC), and other malignancies, with a clinical implications on the applicability in clinical medical strategy. Finally, we discuss future directions, such as AI-guided organoid evolution, immune enhanced models, and scalable manufacturing, to deliver a pragmatic roadmap for implementing PDO in functional precision oncology.

## 2 Establishing PDO: methodological foundations

### 2.1 Clinical sample

PDO are generated from different clinical samples, such as resected tumor specimens, needle biopsies, and fluid-based samples such as ascites or pleural effusions. Tissue acquisition and dissociation strategies (enzymatic, mechanical, or a combination), play a critical role in the initial viability and downstream expansion of organoids (1, 11, 27). The success of organoid generation is dependent on tumor type, site of origin, and histological subtype. For example, gastrointestinal malignancies such as colorectal and pancreatic cancers have a organoid formation rates of 70% (12, 27–32), whereas prostate, breast, and certain sarcomas remain challenging due to stromal contamination or low tumor cellularity (33–38). Samples obtained from metastatic lesions are widely used (39), particularly ascitic or pleural fluid, often exhibit higher establishment efficiency, likely reflecting an enrichment in viable epithelial tumor cells and reduced stromal content (40–43). In contrast, core biopsies from early-stage tumors may result in low-purity organoid cultures due to significant contamination with benign epithelial or mesenchymal cells (16). Moreover, intratumoral heterogeneity and laboratory bias can limit the clonal characteristics of the PDO model (44). Therefore, precise annotation of the origin of the tumor, anatomical location, and processing protocols is fundamental to guarantee interpretability and reproducibility across organoid platforms.

### 2.2 Culture medium and signaling requirements

PDO must be maintained under culture conditions that preserve stemness while allowing controlled lineage commitment and long-term genetic stability (45–47). This requires the use of chemically defined basal media supplemented with tissue-specific growth factors and small-molecule modulators, combined with three-dimensional scaffolds and stringent purity control (48). Standard basal formulations are typically based on Advanced DMEM/F-12 supplemented with B27, HEPES, and GlutaMAX™, and are adapted to the molecular features of the tumor of origin (46, 49).

Foundational studies demonstrated that Wnt signaling is central to intestinal stem cell maintenance and long-term organoid expansion, achieved through supplementation with Wnt3a and R-spondin-1, BMP inhibition via Noggin, and mitogenic stimulation by EGF (10, 40). Subsequent refinements introduced additional components, including gastrin, nicotinamide, and small-molecule inhibitors such as A83-01 (TGF-β pathway inhibition), SB202190 (p38 MAPK inhibition), and Y-27632 (ROCK inhibition), to enhance proliferation and resistance to cellular stress (47, 48, 50).

Importantly, tumors harboring constitutive pathway activation may not require exogenous ligand supplementation. For example, APC-mutant colorectal cancer organoids frequently proliferate independently of Wnt and R-spondin, whereas tumors with activated EGFR/HER2 signaling can be cultured in the absence of EGF (12, 51). Such ligand dependencies can function as functional biomarkers, as illustrated by the requirement for neuregulin-1 (NRG1) in ERBB3/4-driven breast cancer organoids (35).

### 2.3 Extracellular matrix and three-dimensional support

Organoid morphogenesis requires embedding in a permissive three-dimensional extracellular matrix (ECM). Matrigel^®^, a laminin-rich basement membrane extract, remains the most widely used scaffold and was instrumental in early organoid systems (52, 53). However, its batch variability, undefined composition, and regulatory limitations have prompted the development of alternative matrices (54). Natural hydrogels, including collagen I and fibrin–laminin systems, as well as decellularized human ECM, provide biologically relevant microenvironments (55–59). Fully synthetic matrices based on polyethylene glycol (PEG) or dextran allow precise control over stiffness, degradability, and ligand presentation, improving reproducibility and clinical compatibility (60, 61). Defined fibrin–laminin hydrogels have demonstrated robust support for long-term epithelial organoid culture under GMP-compatible conditions, facilitating translational applications (62).

### 2.4 Purity control and morphological validation

Contamination by non-neoplastic epithelial or stromal cells represents a significant confounder in PDO cultures, particularly in early-stage tumors or specimens adjacent to normal tissue (34, 48). Morphologically, tumor organoids recapitulate glandular, cribriform, or solid architectures observed in their tumors of origin (63). Histological and immunohistochemical validation, including lineage markers (CK20, CDX2), proliferation indices (Ki-67), tumor suppressor status (p53), and receptor profiling (HER2, ER, PR), is routinely employed to confirm phenotypic fidelity before functional analyses (64).

### 2.5 Expansion, genetic stability, and biobanking

PDO typically sustain long-term expansion over 10–30 passages, supporting pharmacological and genomic studies (65). Nevertheless, extended culture may introduce selective pressures and clonal drift, underscoring the need for periodic genomic validation through targeted sequencing or single-cell approaches (66). Standard cryopreservation protocols using slow freezing in 10% DMSO and storage in liquid nitrogen preserve viability and functional integrity upon thawing. These advances have enabled the creation of living biobanks that power population-scale pharmacogenomic screening and biomarker discovery efforts (67). Continuous cross-validation with matched primary tumor data remains critical to maintain translational relevance.

## 3 Applications in oncology

### 3.1 Expanding translational impact of PDO across therapeutic modalities

The implementation of PDO across multiple oncological domains has transformed them from static *in vitro* models into dynamic, predictive platforms central to precision medicine. By retaining the architectural organization, mutational landscape, and signaling complexity of their parental tumors, PDO provide functional access to tumor biology that complements genomic profiling. Their applications now span high-throughput pharmacotyping, radiobiology, immuno-oncology, and functional genomics, with emerging integration into microphysiological systems and spatial omics technologies.

### 3.2 Chemotherapy and drug screening

High-throughput drug screening represents one of the most mature applications of PDO technology. Miniaturized 384- and 1536-well formats enable multiplexed screening of thousands of compounds using microliter-scale tissue inputs, coupled with automated imaging and viability readouts that ensure scalability and reproducibility (14, 68).

Multiple prospective and co-clinical studies have shown encouraging concordance between PDO drug sensitivity and patient outcomes. In metastatic colorectal cancer, organoid responses to fluoropyrimidine–oxaliplatin regimens correlate with progression-free survival and radiologic response (12, 68). Similarly, in pancreatic ductal adenocarcinoma (PDAC), PDO pharmacotyping has prospectively stratified responders and non-responders across heterogeneous patient cohorts (69). In breast cancer, organoid-based sensitivity to chemotherapy and targeted agents has been shown to recapitulate clinical responses, particularly in triple-negative disease (35). Notably, early clinical studies have reported high predictive performance, with positive predictive values approaching 80–90% and negative predictive values nearing 100% in selected cohorts (68, 70). Beyond prediction, PDO have provided mechanistic insights into therapeutic resistance.Large-scale combinatorial screening has further identified synthetic lethal interactions, including co-targeting of MEK and ERBB pathways in RAS-mutant tumors (71).

Standardization of culture conditions and assay pipelines has facilitated the transition toward clinical implementation. Large PDO biobanks coupled with pharmacogenomic datasets have enabled systematic drug-response mapping across tumor types, including biliary, liver, endometrial, and head and neck cancers (15, 72–75). Integration with spatial transcriptomics and stromal profiling is further refining drug-response interpretation within the tumor microenvironment, positioning PDO as functional avatars for real-time therapeutic decision-making.

### 3.3 Radiotherapy

PDO technology is increasingly redefining experimental radiobiology by enabling patient-specific prediction of radiation response and systematic identification of radiosensitizing strategies. In rectal cancer, treatment-naïve organoids prospectively stratified patients according to pathological complete response following neoadjuvant chemoradiotherapy (29, 76). However, beyond colorectal cancer, evidence remains more limited and largely preclinical. Studies in glioblastoma and other tumor types suggest that organoid models can capture heterogeneity in radiation response, although prospective validation in clinical cohorts is still lacking (48, 77). PDO further support mechanistic interrogation of radiation response. Collectively, these studies establish PDO as functional biomarkers for radiation sensitivity and as versatile platforms for rational dose escalation and combination therapy design (78).

### 3.4 Immunotherapy

The absence of immune components in conventional epithelial PDO cultures initially limited their applicability in immuno-oncology. However, this limitation has been partially overcome through the development of co-culture systems and air–liquid interface (ALI) models, which preserve or reintroduce tumor–immune interactions. Dijkstra et al. demonstrated that co-culture of tumor organoids with autologous T cells enables the identification and expansion of tumor-reactive lymphocytes, providing a functional platform to study antigen-specific immune responses (79). Neal et al. further developed ALI organoid cultures that retain endogenous immune and stromal components, enabling ex vivo interrogation of immune checkpoint responses (16). These approaches have since been extended to additional tumor types, including lung cancers, although their use remains largely experimental (80). PDO systems have also been used to evaluate engineered immune therapies. For example, CAR-T cell activity has been successfully tested in organoid models, demonstrating tumor-specific cytotoxicity in a 3D context (81). Finally, the integration of PDO with microfluidic systems and tumor-on-chip platforms is enabling more advanced modeling of immune-cell trafficking and tumor–stroma interactions, although these approaches remain at a preclinical stage.

### 3.5 Mechanistic insights

Beyond translational applications, PDO represent powerful systems for modeling cancer biology in a patient-specific context. CRISPR–Cas9 genome editing has enabled stepwise introduction of driver mutations, recapitulating tumor initiation and progression in colorectal and pancreatic organoid models (82, 83). Longitudinal organoid cultures have provided direct insight into clonal evolution and intratumoral heterogeneity, revealing parallel evolutionary trajectories and selection under therapeutic pressure (84). Integration with single-cell transcriptomic approaches has further characterized cellular diversity and lineage hierarchies within tumors. PDO have also been used to study early adaptive responses to therapy, including metabolic rewiring and tumor–stroma interactions, which have been shown to drive endocrine resistance and tumor progression. Transcriptional reprogramming and phenotypic plasticity have been observed following drug exposure, highlighting the emergence of reversible drug-tolerant states that may precede stable resistance (85). These findings establish PDO as experimentally tractable systems for studying tumor evolution and resistance dynamics in real time.

## 4 Tumor-specific evidence

### 4.1 Colorectal cancer

Colorectal cancer (CRC) remains a major therapeutic challenge and a paradigmatic model for precision oncology. Despite multimodal treatment strategies, median overall survival in metastatic CRC (mCRC) has plateaued at approximately 30 months, with most tumors ultimately developing resistance to systemic therapies (86). This therapeutic ceiling, together with the marked inter- and intra-tumoral heterogeneity of CRC, makes the disease particularly suitable for functional precision-oncology approaches based on PDO. The translational relevance of CRC PDO is supported by several converging lines of evidence. First, organoid derivation is technically mature and highly efficient. CRC was the first solid tumor for which robust long-term organoid cultures were established, and current biopsy-based workflows report derivation efficiencies ranging from approximately 60% to 80%, with success rates exceeding 70% in optimized high-throughput platforms (29, 68). Importantly, CRC organoids can typically be established and expanded within 7–14 days, enabling drug-testing timelines compatible with clinical decision-making, particularly in later-line or metastatic settings (87). Second, CRC PDO exhibit high molecular, transcriptomic, and histopathological fidelity to their parental tumors. Comprehensive genomic analyses have demonstrated stable preservation of key driver alterations, including APC, KRAS, TP53, and SMAD4 mutations, as well as conservation of consensus molecular subtypes (CMS1–4), which are associated with distinct biological behaviors and therapeutic vulnerabilities. Organoids also retain intratumoral heterogeneity and clonal architecture, allowing interrogation of evolutionary dynamics and treatment-induced selection pressures (14, 49, 84). Third, a growing body of clinical evidence supports the predictive capacity of PDO-based pharmacotyping in CRC. Landmark studies by Vlachogiannis et al. and Ooft et al. demonstrated that organoid drug-response profiles recapitulate patient sensitivity to standard chemotherapeutic regimens, including fluoropyrimidine–oxaliplatin combinations, as well as targeted therapies such as anti-EGFR agents. Subsequent prospective and co-clinical studies have confirmed significant associations between ex vivo drug-response metrics—including AUC, IC_50_, and growth-rate inhibition—and clinical outcomes, such as radiological response and progression-free survival (29, 44, 68, 88). Across studies, concordance rates between organoid sensitivity and patient response generally range from 70% to over 90%, frequently exceeding the predictive performance of single-gene biomarkers in unselected populations. A growing number of prospective and interventional studies are now incorporating PDO-based pharmacotyping into clinical decision-making frameworks in CRC, spanning assay-guided approaches, adaptive trial designs, and companion diagnostic development (**Table 1**). Beyond response prediction, CRC PDO platforms are enabling systematic exploration of therapeutic vulnerabilities and resistance mechanisms. High-throughput pharmacological screening of large organoid biobanks has identified subtype-specific drug sensitivities and synthetic-lethal interactions, including vulnerabilities associated with RAS/RAF pathway activation, DNA damage response defects, epigenetic dependencies, and metabolic rewiring. Notably, combinatorial strategies targeting adaptive signaling feedback loops, such as dual inhibition of MEK and EGFR/ERBB signaling. have demonstrated efficacy in KRAS-mutant colorectal cancer organoids by overcoming pathway reactivation mechanisms (99). Beyond MAPK pathway targeting, organoid-based screens have uncovered additional therapeutic vulnerabilities, including epigenetic regulators (e.g., chromatin remodeling and histone modification pathways), stemness-associated signaling (Wnt/β-catenin and LGR5+ cell populations), and tumor–microenvironment dependencies that regulate cellular dormancy and resistance states (45, 49).

**Table 1:** Clinical trials on PDO–guided therapy in colorectal cancer.

Trial	Phase/design	N	Endpoint class	Treatment class	PDO metric	Evidence type
Ooft et al., 2019 (68)	Prospective correlation	35	Response (RECIST)	Chemotherapy	Classification (PPV/NPV)	Predictive (validated)
NCT03577808 – Yao et al., 2020 (29)	Co-clinical LARC	90	Response (TRG)	Chemoradiotherapy	Classification (accuracy)	Predictive (validated)
Narasimhan et al., 2020 (89)	Prospective, peritoneal mets	28	Response (clinical)	Multiple (screening)	Operational (take rate)	Feasibility
NICHE – NCT03026140 (90)	Exploratory neoadj. ICI	40	Response (pathological)	Immunotherapy	None	Non-predictive
TUMOROID study 2021 (68)	Observational mCRC	32	Response (RECIST)	Chemotherapy	Drug response (GR-AUC)	Predictive (validated)
APOLLO – NCT03251612 (91)	Phase II mCRC	34 treated	Survival (PFS)	Chemotherapy	None	Interventional
NCT04755907 – Sun et al., 2023 (88)	Prospective validation	40	Response (clinical)	Chemotherapy	Correlation (r)	Predictive (validated)
NCT04996355 “Organoids-on-a-chip” (92)	Observational	52	Response (clinical)	Multiple (screening)	Classification (sens/spec)	Predictive (ongoing)
NCT05304741 Chongqing (ClinicalTrials.gov) (93)	Observational	50 (est.)	None	Multiple (screening)	Operational (TAT)	Feasibility
NCT04906733 Cetuximab-PDO (ClinicalTrials.gov) (94)	Observational	80	Response (ORR)	Targeted therapy (EGFR)	Classification (sensitivity)	Predictive (ongoing)
NCT06012734 LB-100 + atezolizumab (ClinicalTrials.gov) (95)	Phase Ib/II	≤37	Response (ORR)	Combination (immunotherapy-based)	Biological (immune correlates)	Interventional
NCT05401318 TargetCRC (ClinicalTrials.gov) (96)	Observational	40	None	Multiple (screening)	Operational + correlation	Feasibility
NCT04279509 – Selecting Chemo Using PDO (ClinicalTrials.gov) (97)	Single-center Prospective	12 (Stage I)	None	Chemotherapy	Operational (feasibility)	Feasibility
NCT04371198 – Patient-Derived Organoids for Rectal Cancer (ClinicalTrials.gov) (98)	Feasibility/diagnostic	20	Response (clinical)	Radiotherapy	Operational (growth success)	Feasibility
NCT05384184 – Biobank CRC liver mets (ClinicalTrials.gov) (48)	Observational feasibility	TBD	None	Not applicable	Operational (take rate)	Feasibility

Clinical endpoints were grouped into standardized categories (response, survival, disease control, or feasibility), with specific measures indicated in parentheses (e.g. RECIST, pCR, PFS). Treatment class refers to the type of therapy evaluated (e.g. chemotherapy, targeted therapy, immunotherapy, or multi-drug screening platforms). PDO-derived metrics were classified into drug-response metrics (e.g. AUC, GR-AUC), classification performance (e.g. accuracy, sensitivity/specificity), correlation measures (e.g. Pearson r), operational metrics (e.g. take rate, turnaround time), or biological readouts. Evidence type reflects whether studies demonstrated predictive value, feasibility, or interventional application. RECIST, Response Evaluation Criteria in Solid Tumors; TRG, tumor regression grade; ORR, objective response rate; PFS, progression-free survival; MDT, multidisciplinary tumor board; AUC, area under the curve; GR-AUC, growth rate–adjusted area under the curve; TAT, turnaround time.

Importantly, multiple clinical study designs are now integrating PDO into therapeutic decision-making frameworks in CRC. These include assay-guided “n-of-one” approaches, adaptive umbrella trials incorporating organoid-based pre-screening, companion diagnostic development programs, and prospective observational studies evaluating concordance between organoid response and patient outcomes. Collectively, these efforts are shifting CRC organoids from a correlative research tool toward a clinically actionable platform.

Despite these advances, challenges remain. Methodological heterogeneity in culture conditions, assay readouts, and response metrics continues to limit cross-study comparability. Standardization of protocols and prospective validation in interventional trials will be essential to fully establish the clinical utility of CRC PDO. Nonetheless, the accumulated evidence positions colorectal cancer as one of the most mature and clinically validated settings for the implementation of functional precision oncology based on PDO.

### 4.2 Pancreas cancer

Pancreatic cancer remains one of the most lethal solid malignancies, with a 5-year overall survival rate of approximately 12% (100). Despite advances in molecular stratification and combination chemotherapy, therapeutic outcomes remain modest, largely due to extensive genomic instability, phenotypic plasticity, and early development of drug resistance (101). Consequently, PDAC represents a paradigmatic setting in which functional precision-oncology approaches based on PDO may offer substantial clinical added value.

The translational relevance of PDAC PDO is supported by several converging lines of evidence. First, organoid derivation is now technically mature and compatible with routine clinical workflows. Following the pioneering work by Boj et al., which established long-term expansion of human and murine pancreatic ductal epithelium, multiple groups have optimized protocols for both resected specimens and minimally invasive biopsies (102). Current studies report derivation efficiencies of 50–80% from surgical material and ≥60% from EUS-FNB samples, with take-rates exceeding 80% in high-volume centers (103). Importantly, standardized miniaturized assays enable pharmacological profiling within 7–10 days, allowing timely integration into neoadjuvant and first-line treatment decisions (12).

Second, PDAC PDO exhibit high molecular, transcriptomic, and histopathological fidelity to their parental tumors. Whole-exome and targeted sequencing analyses have consistently demonstrated conservation of core driver alterations, including KRAS, TP53, CDKN2A/B, and SMAD4 mutations. Transcriptomic profiling further confirms preservation of the major PDAC subtypes, notably the “classical” and “basal-like” programs, which are associated with distinct therapeutic vulnerabilities (104). Spatial organization, cellular differentiation states, and clonal architecture are likewise retained over serial passages, supporting the suitability of these models for longitudinal and evolutionary studies (105).

Third, an expanding body of clinical evidence supports the predictive capacity of PDO-based pharmacotyping. Early correlative studies by Tiriac et al. and Vlachogiannis et al. demonstrated that organoid drug-response profiles recapitulate patient sensitivity to standard regimens, including gemcitabine- and platinum-based combinations (12, 14). Subsequent prospective cohorts have confirmed these observations, reporting significant associations between ex vivo metrics (AUC, IC_50_, GR scores) and radiological or pathological responses (70). Collectively, reported concordance rates range from 70% to over 90%, frequently exceeding the predictive performance of single-gene biomarkers in unselected populations. Several prospective and observational studies have now evaluated the clinical feasibility and utility of PDO-guided therapy in PDAC (**Table 2**). Beyond response prediction, PDO platforms are enabling systematic exploration of novel therapeutic vulnerabilities in PDAC. High-throughput screening of annotated organoid biobanks has identified context-dependent synthetic-lethal interactions involving chromatin remodelers, DNA damage response pathways, and metabolic regulators (105). For instance, ARID1A-deficient models display enhanced sensitivity to ATR inhibition and SRC-family kinase blockade, while KRAS G12D–mutant organoids exhibit synergistic responses to combined KRAS and EGFR targeting (70). Moreover, organoid-based studies have facilitated rapid preclinical evaluation of emerging agents, including covalent and non-covalent KRAS inhibitors, SHP2 modulators, and ferroptosis inducers. Integration with CRISPR–Cas9 screening, single-cell transcriptomics, and phosphoproteomic profiling is further refining biomarker discovery and resistance modeling, thereby supporting rational combination strategies tailored to individual tumors (70).

**Table 2:** Clinical trials on PDO–guided therapy in pancreas cancer.

Trial/year	Phase/design	N	Endpoint class	Treatment class	PDO metric	Evidence type
Tiriac et al., 2018 (12)	Exploratory	55	Response (RECIST)	Chemotherapy	Drug response (AUC)	Predictive (validated)
Wansch et al., 2025 (106)	Prospective translational	13	Response (clinical)	Multiple (screening)	Classification (accuracy)	Predictive (validated)
Matsumoto et al. 2024 (107)	Prospective cohort	36–50	Survival/Response	Multiple	Biological	Predictive (trend)
HOPE pilot (Grossman 2022) (108)	Prospective feasibility	76	Disease control	Multiple (screening)	Classification (sens/spec)	Predictive (validated)
Demyan 2022 (109)	Prospective NAT	117	Response (pathological)	Chemotherapy	Classification (accuracy) + operational (TAT)	Predictive (validated)
Boilève et al., 2024 (70)	Prospective	87	Survival/Response (PFS/ORR)	Multiple (screening)	Operational (TAT)	Interventional
NCT04931394 (Shanghai) (110)	Phase III RCT	200	Survival (DFS)	Chemotherapy	Correlation	Predictive (ongoing)
Kim 2024 (EUS-FNB avatar) (111)	Prospective technical	113	Response (clinical)	Multiple	Correlation (r) + operational	Predictive (validated)
OPT-I (Wilmink 2022) – NCT03500068 (112)	Feasibility	30	None	Chemotherapy	Operational (take rate, TAT)	Feasibility
Sharick JT 2020 (113)	Exploratory	17	Response (drug response)	Multiple	Biological	Predictive (exploratory)
Pharmacotyping of Pancreatic PDO – NCT05196334 (2022) (114)	Observational	88	Response (RECIST change)	Multiple	Correlation	Predictive (ongoing)
Prediction Platform – Neoadjuvant NCT04777604 (Samsung 2021) (115)	Prospective NAT	300	Response (pCR)	Chemotherapy	Correlation	Predictive (ongoing)
Prediction Platform – Adjuvant NCT04736043 (Samsung 2021) (116)	Prospective adjuvant	300	Survival (DFS)	Chemotherapy	Classification (accuracy)	Predictive (ongoing)
Organoid-Guided Chemotherapy NCT04931381 (Changhai 2021) (117)	Phase III RCT	100	Survival (PFS)	Chemotherapy	None	Interventional
Li et al., 2025 (118)	Exploratory	—	None	Not applicable	Biological (multi-omics)	Exploratory
Dong et al., 2025 (119)	Exploratory	—	None	Not applicable	Drug response (dataset)	Exploratory

Clinical endpoints were grouped into standardized categories (response, survival, disease control, or feasibility), with specific measures indicated in parentheses (e.g. RECIST, pCR, PFS). Treatment class refers to the type of therapy evaluated (e.g. chemotherapy, targeted therapy, immunotherapy, or multi-drug screening platforms). PDO-derived metrics were classified into drug-response metrics (e.g. AUC, GR-AUC), classification performance (e.g. accuracy, sensitivity/specificity), correlation measures (e.g. Pearson r), operational metrics (e.g. take rate, turnaround time), or biological readouts. Evidence type reflects whether studies demonstrated predictive value, feasibility, or interventional application. RECIST, Response Evaluation Criteria in Solid Tumors; pCR, pathological complete response; ORR, objective response rate; PFS, progression-free survival; DFS, disease-free survival; MDT, multidisciplinary tumor board; AUC, area under the curve; TAT, turnaround time.

### 4.3 Breast cancer

Breast cancer (BC) is the most frequently diagnosed malignancy worldwide and remains the leading cause of cancer-related mortality among women (120). Although the overall 5-year survival across stages approaches 90%, outcomes for patients with *de novo* metastatic disease remain poor (<30%), reflecting persistent biological heterogeneity, therapeutic resistance, and limited durability of targeted interventions (121, 122).

In hormone receptor-positive BC, intrinsic and acquired resistance to tamoxifen, fulvestrant, aromatase inhibitors, and CDK4/6 inhibitor-based regimens remains a major clinical challenge. BC-PDOs have emerged as valuable functional models for investigating these resistance mechanisms while preserving the genomic and phenotypic characteristics of the original tumors (78, 123). In particular, PDOs retain clinically relevant ESR1 mutations, including hotspot variants such as Y537S and D538G, enabling the study of ligand-independent estrogen receptor signaling and the evaluation of novel endocrine strategies, including next-generation selective estrogen receptor degraders (SERDs). Likewise, comprehensive PDO biobanks have demonstrated faithful preservation of metastatic and treatment-resistant disease, supporting personalized drug testing and biomarker discovery ( (124)).

Beyond ESR1 mutations, PDOs have increasingly been used to investigate resistance to CDK4/6 inhibitors, revealing adaptive signaling programs that may be therapeutically exploited, as well as activation of the PI3K/AKT/mTOR pathway, a major mechanism underlying acquired endocrine resistance and an important target for combination therapies (125). Emerging evidence also supports the application of PDOs in HER2-low breast cancer, where organoid-based drug testing may facilitate the assessment of responses to novel HER2-targeted agents and antibody–drug conjugates despite the absence of HER2 amplification (126). By contrast, dedicated studies addressing ESR1 fusion-driven breast cancers in PDO models remain limited, and evidence regarding tumor microenvironment-mediated endocrine resistance is still emerging. Nevertheless, obesity-associated inflammatory cytokines, adipokines, growth factors, and lipid mediators are increasingly recognized as modulators of estrogen receptor signaling and endocrine responsiveness (43, 127–129). The integration of PDOs with stromal or adipocyte co-culture systems, as well as emerging organoid-on-chip technologies, represents a promising strategy to better recapitulate the tumor microenvironment and investigate how microenvironmental cues contribute to endocrine resistance and tumor progression, although further validation is needed before these complex models can be routinely incorporated into functional precision oncology workflows (130–132).

The BC features make BC a clinically relevant setting for functional precision-oncology strategies based on PDO. The translational relevance of breast cancer PDO is supported by several converging lines of evidence. First, organoid derivation is now technically robust and compatible with clinical workflows. Following the pioneering work by Sachs et al., which established long-term culture of primary and metastatic breast tumors as organoids (35), subsequent methodological refinements have enabled derivation efficiencies of 70–90% from surgical specimens and ≥80% from core biopsies (124). Most cultures reach drug-testing readiness within 7–10 days, allowing timely integration into neoadjuvant and metastatic treatment planning (35, 133).

Second, breast cancer PDO exhibit high molecular, transcriptomic, and phenotypic fidelity to their parental tumors. Comprehensive profiling studies have demonstrated consistent preservation of hormone receptor status (ER, PR), HER2 amplification, intrinsic molecular subtypes (Luminal A/B, HER2-enriched, triple-negative), and recurrent driver mutations, including PIK3CA, TP53, and ESR1 (35, 124). Functional drug responses *in vitro* closely mirror those observed in matched patient-derived xenografts and clinical cohorts, and are supported by mechanistic studies highlighting hormone signaling, metabolic crosstalk, and microenvironment-driven resistance pathways (36, 124).

Third, an expanding body of clinical evidence supports the predictive capacity of PDO-based pharmacotyping. Early exploratory and correlative studies demonstrated that organoid drug-response profiles recapitulate patient sensitivity to chemotherapy, endocrine therapy, and HER2-directed regimens (35). Subsequent retrospective and prospective cohorts have confirmed significant associations between ex vivo metrics (AUC, IC_50_, GR scores) and radiological or pathological outcomes. Across studies, reported concordance rates range from approximately 70% to over 90%, frequently exceeding the predictive performance of single-gene biomarkers in unselected populations (14, 44, 133). Building on these findings, multiple phase I/II and phase III interventional trials are currently evaluating organoid-guided therapy selection against physician’s choice regimens in advanced and HER2-positive breast cancer (**Table 3**). Together, these efforts mark the transition from feasibility and correlative validation toward regulatory-grade clinical testing.

**Table 3:** Clinical trials on PDO–guided therapy in breast cancer.

Trial/year	Phase/design	N	Endpoint class	Treatment class	PDO metric	Evidence type
Sachs et al., 2018 (35)	Exploratory library	99	Response (subtype-specific)	Multiple	Drug response (AUC)	Predictive (validated)
Guillen/Welm 2022 (124)	Resource cohort	168	Response (in vivo)	Multiple	Correlation	Predictive (validated)
Lin et al., 2023 (134)	Retrospective metastatic cohort	58	Response (ORR)	Multiple	Classification (response enrichment)	Predictive (validated)
NCT04131881 (135)	Prospective observational (neoadjuvant)	120	Response (pCR)	Chemotherapy	Classification (accuracy) + operational (TAT)	Predictive (validated)
NCT04750122 (136)	Phase I/II	46	Response (pCR)	Targeted therapy (HER2)	Operational	Feasibility
NCT06268652 (90)	Phase III RCT	252	Survival (PFS)	Multiple	None	Interventional
NCT06102824 (137)	Phase II randomised	252	Response (ORR), QoL	Targeted therapy (HER2)	None	Interventional
NCT06438055 (138)	Interventional single-arm	40	Response (RECIST)	Combination	Correlation	Predictive (ongoing)
NCT06468124 (139)	Prospective pilot	20	Survival	Multiple	Correlation	Predictive (ongoing)
NCT06459791 (140)	Prospective technical	30	None	Not applicable	Operational (TAT) + correlation	Feasibility
Przanowska et al., 2024 (133)	Prospective translational	—	None	Not applicable	Biological	Exploratory
Zhang et al., 2025 (141)	Case-based translational	1	Response (clinical)	Multiple	Correlation	Predictive (case-level)

Clinical endpoints were grouped into standardized categories (response, survival, disease control, or feasibility), with specific measures indicated in parentheses (e.g. RECIST, pCR, PFS). Treatment class refers to the type of therapy evaluated (e.g. chemotherapy, targeted therapy, immunotherapy, or multi-drug screening platforms). PDO-derived metrics were classified into drug-response metrics (e.g. AUC, GR-AUC), classification performance (e.g. accuracy, sensitivity/specificity), correlation measures (e.g. Pearson r), operational metrics (e.g. take rate, turnaround time), or biological readouts. Evidence type reflects whether studies demonstrated predictive value, feasibility, or interventional application. RECIST, Response Evaluation Criteria in Solid Tumors; pCR, pathological complete response; ORR, objective response rate; PFS, progression-free survival; QoL, quality of life; MDT, multidisciplinary tumor board; AUC, area under the curve; TAT, turnaround time.

### 4.4 Other cancers: extending functional organoid platforms across solid and hematological malignancies

#### 4.4.1 Prostate cancer

Prostate cancer organoid modeling has evolved substantially since the first long-term cultures of metastatic castration-resistant prostate cancer (CRPC) were described (34). Refinements in androgen-regulated and R-spondin–modulated culture conditions now allow sustained expansion of metastatic and treatment-resistant disease (Beshiri et al., 2018; Tuveson e Clevers 2019). Prostate PDO preserve androgen receptor (AR) dependency, ETS rearrangements (e.g., *TMPRSS2–ERG*), DNA repair defects (*BRCA2*, *ATM*) and neuroendocrine differentiation emerging under AR-targeted therapy. Functionally, these models have been used to predict response to AR signaling inhibitors and PARP inhibitors in homologous recombination–deficient subsets, as well as to model lineage plasticity and therapy-induced transdifferentiation. Integration with single-cell RNA sequencing has revealed coexistence of luminal, basal-like, and neuroendocrine-like states within individual patients, reinforcing the value of functional readouts beyond static genomics (142, 143) Although prospective interventional trials remain limited, prostate PDO are emerging as a promising adjunct in metastatic CRPC stratification frameworks (144).

#### 4.4.2 Glioblastoma and central nervous system tumors

Glioblastoma (GBM) represents one of the most treatment-refractory solid tumors, characterized by intratumoral heterogeneity, hypoxia-driven plasticity, and profound microenvironmental influence. Brain tumor organoids and tumor–brain assembloids have demonstrated the capacity to recapitulate hypoxic gradients, stem-like niches, invasive growth patterns, radiation response heterogeneity (77, 145, 146). Moreover, GBM-derived organoids retain genomic hallmarks such as *EGFR* amplification, *PTEN* loss, and *TP53* mutations, while preserving transcriptional states reflective of proneural and mesenchymal subtypes (77, 145, 146). Emerging data suggest concordance between ex vivo organoid sensitivity to temozolomide and radiotherapy and patient-specific clinical outcomes, although validation remains exploratory (147). The integration of microfluidic perfusion systems and immune components may further enhance the translational relevance of GBM organoids.

#### 4.4.3 Lymphomas and hematological malignancies

The therapeutic landscape of aggressive B-cell lymphomas has expanded beyond conventional immunochemotherapy to include molecularly targeted agents and CD19-directed immunotherapies (148–153). Although organoid platforms were initially optimized for epithelial tumors, recent advances demonstrate that 3D organotypic systems can be adapted to hematological malignancies when appropriate stromal and immune support is incorporated. Diffuse large B-cell lymphoma (DLBCL), follicular lymphoma, and mantle cell lymphoma have been successfully maintained in 3D matrices enriched with mesenchymal stromal cells, partially reconstructing germinal center–like architecture (154, 155) These lymphoma organotypic systems preserve molecular subtype signatures (GCB vs ABC DLBCL), MYC, BCL2, and BCL6 rearrangements, B-cell receptor (BCR) signaling dependency and NF-κB activation states. Functional assays performed in 3D culture demonstrate heterogeneous sensitivity to BTK inhibitors, PI3K inhibitors, and BCL2 antagonists, underscoring the importance of functional over purely genomic stratification.

## 5 Limitations and current challenges of PDO

### 5.1 Biological and technical limitations affecting clinical implementation

**Figure 1:**
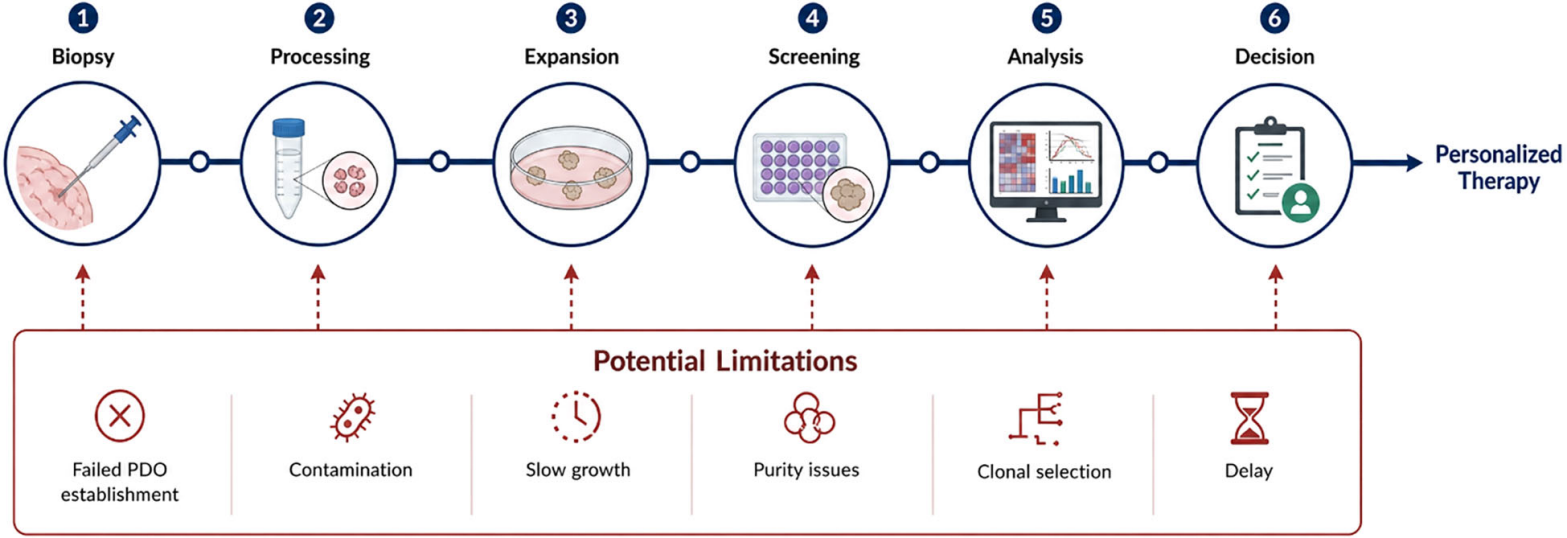
Clinical timeline and practical challenges of PDO-guided functional precision oncology. Following tumor biopsy, PDO establishment, expansion, and drug screening are integrated with clinical and molecular data to support personalized treatment decisions. The timeline may vary according to tumor type and laboratory workflow.

The transition of PDO from experimental platforms to clinically actionable tools represents one of the most critical inflection points in functional precision oncology. A practical limitation concerns the successful establishment of PDO cultures. Organoid derivation rates vary substantially across tumor types, specimen quality, and sampling procedures, with failures resulting from low tumor cellularity, microbial contamination, tissue necrosis, overgrowth of non-neoplastic epithelium, or insufficient proliferative capacity. Even when cultures are successfully established, slow expansion kinetics may preclude clinically actionable drug testing within the therapeutic decision window. This transition is contingent upon rigorous analytical standardization, harmonized regulatory frameworks, and demonstrable health-economic value. A major barrier to clinical translation remains the substantial inter- and intra-laboratory variability inherent to PDO workflows. Differences in tissue procurement, dissociation protocols, extracellular matrix composition, media formulations, passage number, and assay readouts can profoundly influence organoid establishment rates, clonal composition, and drug-response phenotypes (156–158) (**Figure 1**).

Even lower variations in growth factor concentrations or matrix stiffness have been shown to alter lineage commitment and signaling dependencies, thereby affecting pharmacological outputs. Consequently, the lack of standardized operating procedures (SOPs) continues to limit reproducibility and cross-study comparability. While recent advances in automated and miniaturized high-throughput platforms have improved scalability and reduced operator-dependent variability, consensus on standardized pharmacodynamic endpoints remains incomplete. Metrics such as area under the curve (AUC), IC_50_, and growth-rate (GR) inhibition provide complementary but not interchangeable measures of drug response (159–162). Importantly, GR-based metrics have been proposed to correct for confounding effects of proliferation rates, yet their adoption in clinical-grade pipelines is still heterogeneous. A unified framework integrating viability, growth kinetics, and phenotypic readouts, potentially supported by high-content imaging features, will be required to ensure robust and clinically interpretable outputs (159, 163).

Central to analytical validation is the definition and regulatory qualification of critical quality attributes (CQAs) (156). These include:

- genomic concordance with the parental tumor, assessed through targeted or whole-exome sequencing;
- transcriptomic and epigenetic stability across passages;
- preservation of intratumoral heterogeneity;
- reproducibility of dose–response relationships across technical replicates and timepoints.

Another important aspect of PDO validation is the availability of appropriate biological controls. The use of autologous healthy organoids derived from adjacent non-tumoral tissue may help distinguish tumor-specific vulnerabilities from tissue-specific drug responses. Likewise, autologous peripheral blood mononuclear cells (PBMCs) are often required as germline controls for genomic analyses such as whole exome sequencing (WES). Although the integration of these matched controls increases the complexity of PDO workflows, it may substantially improve data interpretation and biological relevance. Beyond these molecular parameters, emerging evidence suggests that functional fidelity, so the ability of PDO to recapitulate patient-specific therapeutic responses, should be considered a core CQA. This shifts the paradigm from descriptive validation toward predictive validation, aligning PDO platforms with regulatory expectations for companion diagnostics. Prospective interventional trials incorporating PDO-guided treatment allocation are now redefining the evidentiary landscape. In several type of cancer described above and reasumed in **Tables 1**–**3**, early studies have demonstrated that PDO-informed therapeutic selection can predict treatment response with clinically meaningful accuracy, often outperforming genomic biomarkers in heterogeneous disease contexts. These studies mark a transition from retrospective concordance analyses toward outcome-driven validation, a prerequisite for regulatory approval. Despite recent advances that have shortened organoid establishment and drug-testing workflows, turnaround time remains a critical determinant of clinical applicability. PDO-guided pharmacotyping is likely to be most informative in settings where treatment decisions can accommodate ex vivo testing, such as metastatic disease, later-line therapy selection, or selected neoadjuvant protocols. Conversely, in patients requiring immediate treatment initiation, delays associated with organoid generation or unsuccessful culture establishment may limit its practical utility.

Regulatory implementation pathways are likely to evolve along multiple, non-mutually exclusive trajectories. PDO-based assays may be approved as companion diagnostics (CDx) linked to specific drugs, incorporated into adaptive clinical trial designs to guide dynamic treatment allocation, or initially introduced as laboratory-developed tests (LDTs) within certified clinical laboratories (164–166). Notably, both the European Medicines Agency and the U.S. Food and Drug Administration have increasingly support the integration of advanced *in vitro* models, including organoids and organ-on-chip platforms, aimed at reducing animal experimentation and accelerating translational pipelines (24, 167). The recent modernization of regulatory frameworks to incorporate real-world evidence and advanced *in vitro* systems further supports the integration of PDO-based assays into clinical decision-making.

**Figure 2:**
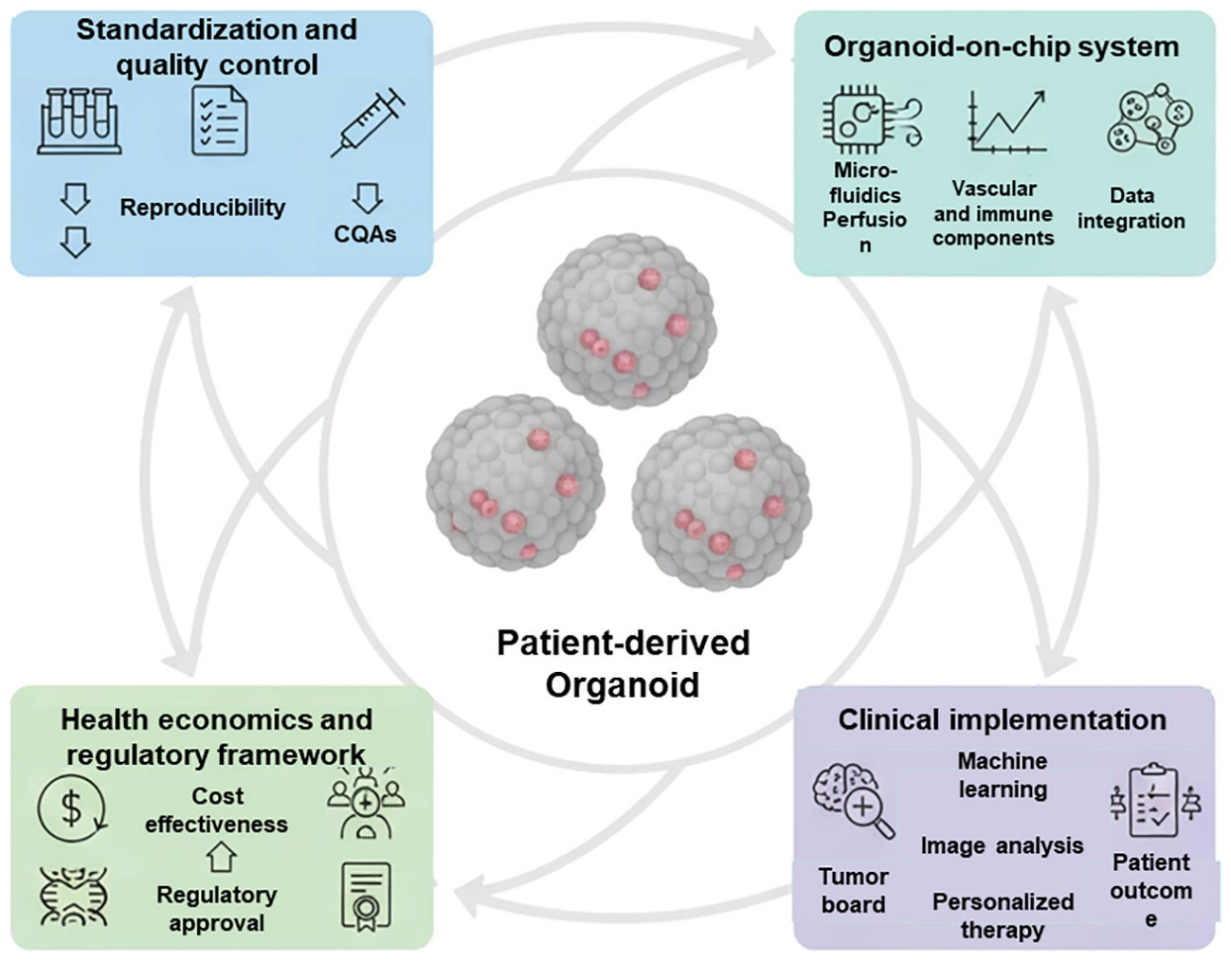
Integrated framework of PDO platforms in precision oncology.

Parallel to regulatory validation, health-economic considerations will be for large-scale implementation. PDO derivation and pharmacotyping entail non-negligible upfront costs, including infrastructure, specialized personnel, and assay standardization (**Figure 2**). However, these costs must be contextualized within the broader oncology care continuum. By enabling early identification of ineffective therapies, PDO-guided approaches have the potential to reduce unnecessary toxicity, avoid futile treatment lines, and optimize therapeutic sequencing (168). Preliminary modeling studies suggest that even modest improvements in response rates or progression-free survival may translate into substantial cost savings at the health-system level, particularly in high-burden malignancies such as colorectal and pancreatic cancer (169, 170).

To support reimbursement and policy adoption, prospective clinical trials should incorporate cost-utility analyses, quality-adjusted life years (QALYs), and budget impact models as predefined endpoints. Importantly, economic evaluations must also consider indirect benefits, including reduced hospitalization, improved quality of life, and more efficient allocation of healthcare resources. The development of standardized reimbursement frameworks will likely depend on demonstrating not only clinical validity but also clinical utility and cost-effectiveness in real-world settings.

An important limitation of PDO-based functional precision oncology is that ex vivo drug sensitivity does not invariably translate into clinical response. Although multiple studies have demonstrated encouraging concordance between pharmacotyping results and patient outcomes, several biological and technical factors may reduce predictive accuracy. Intratumoral heterogeneity and sampling bias may lead to the establishment of organoids that incompletely represent the full clonal landscape of the tumor. Moreover, selective pressures during *in vitro* culture can favor the expansion of specific subclones, potentially altering the original cellular composition. Conventional PDO cultures also lack many components of the tumor microenvironment, including stromal, vascular, and immune cells, which are known to influence treatment response. Finally, temporal divergence between tissue sampling and therapy initiation may become relevant in rapidly evolving or heavily pretreated cancers, where additional molecular changes can emerge between biopsy acquisition and clinical decision-making. These considerations highlight that PDO pharmacotyping should currently be interpreted as a complementary tool to genomic, pathological, and clinical information rather than a standalone predictor of therapeutic efficacy.

PDO are embedded within a dynamic ecosystem integrating standardization strategies, organoid-on-chip systems, and artificial intelligence–driven analytics. These components collectively enable improved reproducibility, physiological fidelity, and predictive modeling, supporting clinical implementation and personalized therapeutic decision-making within a learning healthcare system.

### 5.2 Organoid-on-chip and microphysiological systems

**Figure 3:**
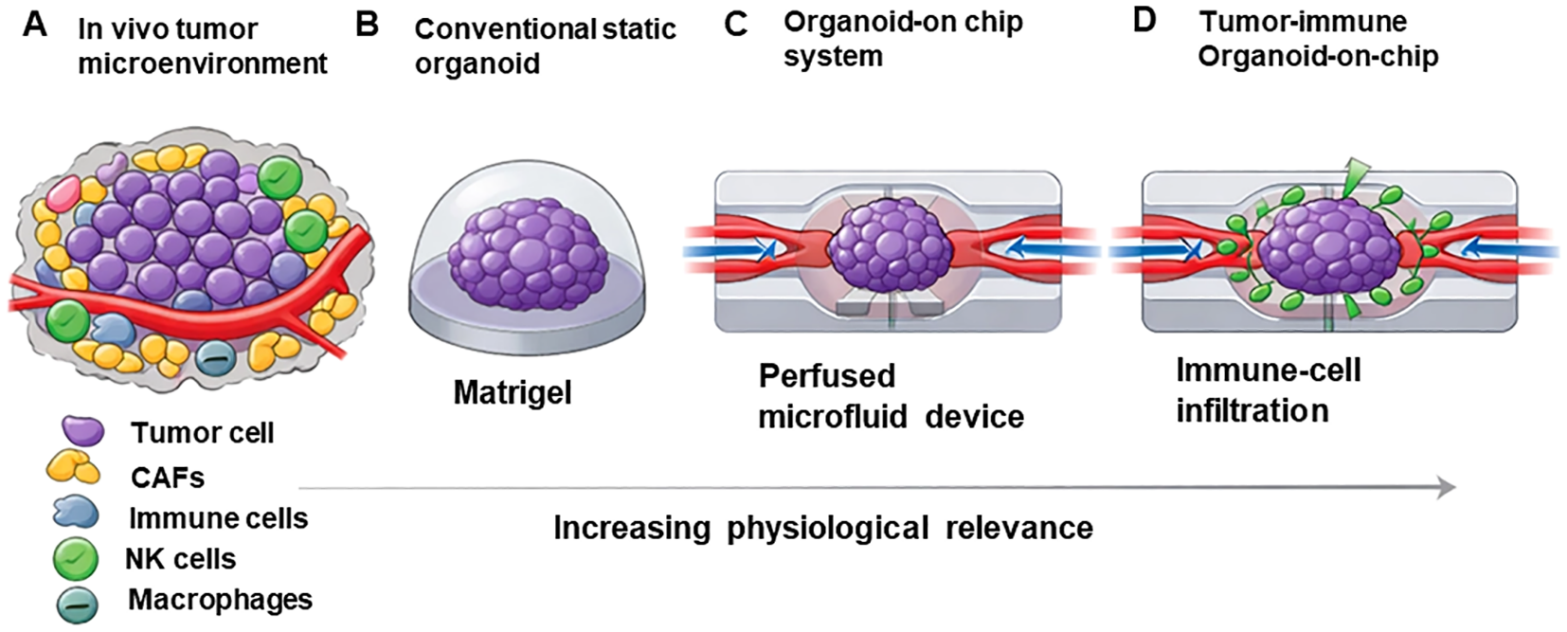
Organoid-on-chip platforms recapitulate key features of the tumor microenvironment. **(A)** Native in vivo tumor microenvironment. **(B)** Conventional static organoid embedded in Matrigel. **(C)** Perfused organoid-on-chip system. **(D)** Tumor–immune organoid-on-chip model enabling immune cell infiltration and enhanced physiological relevance.

Despite their transformative potential, conventional PDO cultures remain constrained by their largely static architecture and incomplete recapitulation of *in vivo* physiology. Standard three-dimensional matrices do not adequately reproduce vascular perfusion, biomechanical cues, oxygen and nutrient gradients, or the spatiotemporal pharmacokinetics of drug exposure. Moreover, they only partially capture the complexity of the tumor microenvironment (TME), including stromal, vascular, and immune components that critically influence tumor progression and therapeutic response (**Figure 3**).

A major advance has been the development of perfused and vascularized organoid-on-chip platforms. In 2024, Quintard and colleagues reported a microfluidic platform capable of integrating functional vascularized organoids-on-chip, showing intravascular perfusion and improved growth, maturation, and tissue function in multiple organoid contexts (171). More broadly, tumor-on-chip systems that reproduce pharmacokinetic drug exposure profiles have shown that dynamic perfusion can alter growth behavior, drug delivery, and treatment response compared with static cultures, supporting their utility for more realistic pharmacodynamic studies (172–179).

In immuno-oncology, microfluidic systems are increasingly used to reconstruct tumor microenvironment and thus spatially organized tumor–stroma–immune interactions. Platforms incorporating endothelial barriers allow the perfusion, transmigration, and infiltration of immune cells into tumor compartments, enabling direct observation of trafficking dynamics and treatment-dependent immune behavior (179–181). For example, a 2024 breast-cancer-on-chip study integrated an endothelial barrier and demonstrated immune-cell transmigration and cytokine monitoring during CAR-T-cell exposure, while other tumor-on-chip models have been used to quantify immune-cell infiltration under chemo-immunotherapy conditions (81). In pancreatic cancer, microfluidic organoid cultures have also been used to test NK-cell-based immunotherapy in combination with a trispecific engager, illustrating the value of dynamic organoid-chip systems for studying immune cytotoxicity in patient-derived material (81, 182–184).

A further step toward clinical translation is the use of multi-organ microphysiological systems (MPS), in which tumor models are linked to liver, gut, bone-marrow, or other organ compartments to capture systemic determinants of efficacy and toxicity (185). Although many of these systems are still being optimized and not all use tumor organoids specifically, the field is clearly moving toward interconnected platforms for studying absorption, metabolism, distribution, off-target toxicity, and metastatic organotropism in a single experimental framework. Recent reviews in toxicology and multi-organ chip design highlight their promise for quantitative pharmacokinetic studies and safety assessment, while large-scale body-on-chip efforts underscore the feasibility of increasingly complex interconnected human-mimetic systems (186–188). Taken together, organoid-on-chip and MPS technologies represent an important bridge between reductionist *in vitro* culture and *in vivo* tumor biology. Their principal strengths lie in dynamic perfusion, multicompartment integration, and the capacity to model tumor–vascular–stromal–immune interactions with greater fidelity than static organoid assays. At the same time, challenges remain in throughput, reproducibility, materials standardization, inter-laboratory transferability, and regulatory qualification, all of which must be addressed before these systems can be broadly embedded into routine precision-oncology workflows.

### 5.3 Artificial intelligence and computational integration

The convergence of PDO technology with artificial intelligence (AI) is rapidly redefining the analytical and predictive capabilities of functional precision oncology. Machine learning and deep learning frameworks are increasingly leveraged to extract high-dimensional phenotypic information from organoid systems, integrating heterogeneous datasets spanning histopathology, genomics, transcriptomics, proteomics, and pharmacological responses (11, 159, 189–193). A major area of progress is image-based phenotyping. Deep-learning pipelines such as CNN-based segmentation and tracking algorithms can quantify organoid number, size, shape, growth kinetics, and viability directly from label-free microscopy, enabling longitudinal and single-organoid analyses at scale (22). Recent studies show that morphology-derived latent features can also capture biologically meaningful inter-tumoral heterogeneity and can be linked to gene-expression programs and drug-response profiles. In this context, image-based readouts are moving from simple descriptive metrics toward functionally informative phenotypes (18, 19, 159, 194, 195).

Beyond descriptive imaging, computational models are increasingly being developed to predict drug sensitivity from organoid-derived data. Network-based machine-learning frameworks have already shown that pharmacogenomic information from colorectal organoids can predict anti-cancer drug efficacy in patients, while newer transfer-learning approaches aim to improve clinical drug-response prediction by combining the biological fidelity of PDO with the scale of large 2D pharmacogenomic datasets. Together, these studies suggest that multimodal computational models may complement, and in some settings outperform, genomics-only stratification, particularly where tumor heterogeneity and non-genetic resistance states play a major role (14, 189, 196, 197).

AI is also beginning to support adaptive experimental design. In organoid and related 3D screening systems, machine learning has been used to prioritize informative regions of large combinatorial drug spaces, refine dosing schedules, and identify more efficient multidrug regimens. Although reinforcement learning per se is not yet a mature standard in PDO workflows, the broader use of active-learning and closed-loop optimization strategies indicates a clear movement toward computationally guided screening rather than exhaustive empirical testing (198, 199).

A particularly promising direction is the development of in silico patient avatars or computational surrogates that integrate PDO pharmacotyping with molecular and clinical variables. At present, this concept is more advanced as a translational framework than as a fully validated clinical tool; however, recent work in pancreatic and colorectal cancer indicates that PDO-informed models can predict response trajectories and treatment benefit more accurately than static molecular profiling alone. In pancreatic ductal adenocarcinoma, ex vivo PDO pharmacotyping has been associated with RECIST response and CA19–9 decline, whereas in metastatic colorectal cancer prospective studies continue to support the clinical predictive value of PDO-based testing (14, 69, 200, 201). Overall, the integration of AI with organoid platforms supports a transition from static, mutation-centered stratification toward a dynamic, functionally informed, and computationally augmented precision-oncology paradigm. The next challenge will be prospective clinical validation, standardization of multimodal pipelines, and rigorous benchmarking across institutions to ensure that AI-assisted organoid readouts become robust decision-support tools rather than purely exploratory research outputs (192, 202).

### 5.4 Toward hospital-integrated organoid platforms

The ultimate clinical impact of PDO technology will depend on its seamless integration into routine healthcare workflows. A pragmatic implementation model envisions embedding PDO pharmacotyping within multidisciplinary tumor boards, forming a closed-loop, learning healthcare system. Emerging prospective and translational studies in colorectal and pancreatic cancers have demonstrated the feasibility of this approach within clinically actionable timelines, with PDO establishment and drug testing typically achievable within approximately 2–4 weeks in optimized settings (14, 68, 69, 200). These timeframes are increasingly compatible with therapeutic decision-making in both neoadjuvant and metastatic contexts, although variability remains depending on tumor type, sample quality, and culture efficiency.

Notably, prospective clinical studies have shown that PDO-based pharmacotyping can correlate with patient response to chemotherapy and targeted agents. In metastatic colorectal cancer, PDO have been reported to predict treatment response with clinically meaningful accuracy, while in pancreatic ductal adenocarcinoma ex vivo drug sensitivity profiles have been associated with radiologic and biomarker responses (68, 69). More recent clinical implementation studies further support the feasibility of integrating organoid testing into real-world clinical workflows (197, 200).

The successful integration of PDO platforms into hospital infrastructures will require several key components: (i) standardized protocols (SOPs) for tissue processing, culture, and drug testing, (ii) robust quality-control and reproducibility pipelines, (iii) regulatory-compliant biobanking frameworks, (iv) AI-assisted multimodal data integration and interpretation, and (v) interoperability with electronic health records (EHRs).

In addition, harmonization across institutions and prospective validation in interventional trials will be essential to ensure clinical reliability and scalability.

Importantly, the implementation of PDO-guided decision-making frameworks may contribute to transforming oncology from a predominantly reactive paradigm into a data-driven, adaptive, and continuously learning system, in which functional drug-response data are iteratively integrated with molecular and clinical information (203, 204). In this context, PDO uniquely bridge the longstanding gap between genotype and phenotype, providing direct measurement of therapeutic vulnerability rather than relying solely on inferred sensitivity.

The convergence of PDO platforms with artificial intelligence and microphysiological systems (organoid-on-chip technologies) further strengthens their translational potential. AI enables scalable interpretation of high-dimensional functional and molecular datasets, while organoid-on-chip systems enhance physiological fidelity by incorporating vascular, stromal, and immune components. Together, these advances position PDO as central elements of next-generation precision oncology pipelines, with the potential to reshape not only individual patient care but also drug development, clinical trial design (e.g., adaptive and basket trials), and health system optimization (14, 203, 204).

## 6 Conclusion

In summary, PDO are emerging as a promising component for functional precision oncology, bridging the gap between molecular profiling and therapeutic response. Their integration with artificial intelligence and microphysiological systems is accelerating the transition toward dynamic, data-driven clinical decision-making. Although challenges in standardization, scalability, and prospective validation remain, the incorporation of PDO-based platforms into hospital workflows may contribute to transforming oncology into a continuously learning healthcare system, ultimately improving patient stratification, therapeutic efficacy, and drug development.
